# Advantage of early orchiopexy for undescended testis: Analysis of testicular growth percentage ratio in patients with unilateral undescended testicle

**DOI:** 10.1038/s41598-017-17825-w

**Published:** 2017-12-12

**Authors:** Chi-Shin Tseng, I-Ni Chiang, Chung-Hung Hong, Yu-Chuan Lu, Jian-Hua Hong, Hong-Chiang Chang, Kuo-How Huang, Yeong-Shiau Pu

**Affiliations:** 1 Department of Urology, National Taiwan University Hospital, Taipei, Taiwan; 2 Graduate Institute of Electronics Engineering, National Taiwan University, Taipei, Taiwan

## Abstract

This study reports the experience of our tertiary referral center and proposes a new indicator, the growth percentage ratio (GPR), for determining the optimal timing of surgical intervention. A retrospective review of boys who underwent orchiopexy for undescended testis from 2001 to 2013 was conducted. We analyzed testicular volumes in different age groups using the UDT to normally descended testis ratio and testicular GPR. A total of 134 boys with unilateral undescended testicle underwent regular ultrasonography follow-up examinations for more than a mean of 3.9 years. Forty-five (33.4%) of them underwent orchiopexy before the age of one year. Orchiopexy at this age resulted in a GPR (2.02 ± 0.40) that was significantly higher than the GPRs in the second (1 < age ≤ 2 years, 1.25 ± 0.13, p = 0.004) and third (age > 2 years, 1.24 ± 0.14 p = 0.008) age groups. The undescended testicle grew faster when orchiopexy was performed before one year of age. Orchiopexy performed within one year from birth significantly accelerates the growth of the UDT, as determined using the GPR, compared to other age groups. The present clinical evidence indicates that orchiopexy should be performed before one year of age.

## Introduction

Cryptorchidism is one of the most common congenital urological diseases. The prevalence of cryptorchidism at birth varies from 2 to 5%^1–3^, and the testes mostly descend during the first 6 months of life^4^. A higher risk of infertility and testicular malignancy associated with failure of testicular descent can be reduced significantly by correction of cryptorchidism^5,6^. Over the past few decades, the recommended age of orchiopexy progressively decreased from 10–15 years in the 1950s^7^ to 5–6 in the 1970s^8^, 2 in the early 1980s^9^, and 1–2 in the 1990s and 2000s^10^. Currently, orchiopexy is recommended between 6 and 12–18 months^11^. However, the optimal timing of surgical intervention is still widely debated. Numerous studied showed that the average age of boys with undescended testis (UDT) at the time of surgery is still above the recommended age^12^.

A major concern with regard to UDT is potential impaired fertility. Since 80–90% of a testis is composed of seminiferous tubules, the volume of the testis is significantly related to the semen profile and testicular function^13^. Measuring the testicular volume and growth is an effective practical method of evaluating the development of the testes. Moreover, monitoring of testicular function is not possible in this quite young age. Most current studies have documented satisfactory testicular growth as long as orchiopexy was performed before two years of age^14^. However, there is a scarcity of studies of testicular outcomes when orchiopexy is performed within the first year of life. To address this issue, we conducted a retrospective review to evaluate testicular growth after early orchiopexy at our institution.

## Materials and Methods

The Institutional review board (IRB) of National Taiwan University Hospital approved this study (IRB #201704024RINA), including patient recruitment, chart review, informed consent and all study methods. All methods were performed in accordance with the relevant guidelines and regulations of the institution. A total of 518 boys who underwent orchiopexy because of UDT (ICD9 752.51) from January 2001 to December 2013 were identified. Patients’ clinical characteristics, concomitant diseases, laterality of the disease, age of orchiopexy, pre-operative and post-operative scrotal ultrasonography results, and intra-operative findings were collected.

The operative procedures were performed by three pediatric surgeons and two general urologists independently. The standard inguinal approach or primary scrotal orchiopexy were performed. Ultrasonography before and after orchiopexy was conducted in 182 patients. The remaining patients, who underwent either a single ultrasonography examination or a physical examination only for monitoring testicular outcomes, were excluded from analysis.

### Definition of undescended testes

Patients who underwent orchiopexy because of retractile testes, gliding testes, and acquired undescended testes were not included. Congenital cryptorchidism was defined as absence of the testicle in the scrotal position since birth. All testes that could not be pulled down to the scrotum were documented as undescended testes. In contrast, a testicle that could be pulled down to the scrotal position and retained in the scrotum was defined as a retractile testis. A testicle that could be pulled down to the scrotal position but retracted when released was defined as a gliding testis. A testis that had previously descended but then “ascended” spontaneously was defined as an acquired undescended testis. A testis that descended from the inguinal canal to the femoral region, perineum, or contralateral scrotum was defined as an ectopic testis.

### Analysis of Testicular Volume and Testicular Growth

We applied high-resolution ultrasonography with a linear-array 7.5 and 10 MHz transducer to measure the length, width, and position of the testicles. A single urologist reviewed the sonography images to confirm the accuracy and quality of the measurement. Testicular volume was calculated using the Hansen formula^15^: testicular volume = 0.52 × length [L] × width [W]^2^.

In this study, growth percentage of a testis was defined as post-operative testicular volume divided by pre-operative testicular volume × 100%. Since growth percentage of a testis depends on follow-up time, we used the growth percentage ratio (GPR), which was defined as growth percentage of the UDT divided by growth percentage of the contralateral normally descended testis (NDT).

We compared GPRs in these different age groups to evaluate the benefit of early orchiopexy. The traditional analytical measure, the UDT to NDT ratio, and testicular atrophy index (TAI) were also determined. The outcomes included testicular volumes after orchiopexy, UDT to NDT ratio, and GPR.

### Statistical Methods

For comparison of continuous variables between two groups and three groups, we used the independent Student T-test and analysis of variance (ANOVA). The Mann-Whitney U test was used for comparison of two independent groups when data were not normally distributed. For comparisons between categorical variables, we used the Chi-square test. A *p* value <0.05 was considered to indicate a statistically significant difference. Analyses were performed using the SPSS statistics software, version 22.0 (IBM Corp, SPSS, Inc., Chicago, IL).

## Results

**Figure 1:**
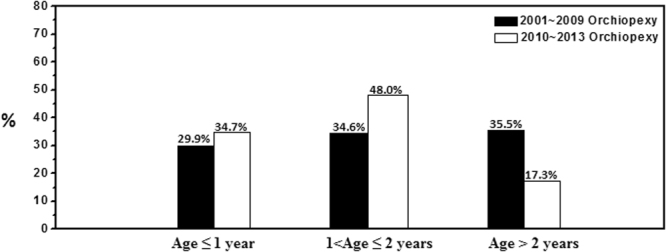
Changes in age at orchiopexy in different time periods.

In this study, orchiopexy for undescended testicles was performed much earlier in the recent years (2010–2013) than in the previous decade (2001–2009) (Fig. 1). The fraction of orchiopexies conducted after the age of 2 years old declined from 35.5 to 17.3%. In contrast, the fraction of early orchiopexies increased from 29.9 to 34.7%. The overall median age at orchiopexy in 182 patients with UDT was 13.9 months (interquartile range (IQR) 11.2–27.3), and the overall median duration of follow-up with scrotal ultrasonography was 33.8 months (IQR 20.1–52.7). The patients were separated into three groups by age at orchiopexy: age ≤ 1 year (group I, n = 58), 1 < age ≤ 2 years (group II, n = 73), and age > 2 years (group III, n = 51). Table 1 lists patient characteristics, follow-up duration, and laterality of undescended testicles for each age group. The proportions of laterality of UDT were similar between group I and group II (p = 0.879) and between group I and group III (p = 0.654).

**Table 1:** Patient characteristics of patients with undescended testis.

Group	I	II	III	Total
	Age ≤1 year	1< Age ≤2 years	Age >2 years	
Patients number	58	73	51	182
Median age (IQR), months	9.2 (7.2–11.6)	14.3 (13.2–17.7)	68.6 (40.9–90.0)	
Follow-up (IQR), months	35.5 (25.4–57.5)	36.6 (21.2–54.8)	28.2 (11.5–50.9)	
Bilateral UDT	13 (22.4%)	15 (20.5%)	20 (39.2%)	48
Left UDT	21 (36.2%)	29 (39.7%)	12 (23.5%)	62
Right UDT	24 (41.4%)	29 (39.7%)	19 (37.3%)	72

A total of 134 (73.6%) patients with a unilateral UDT were further divided into three groups according to age at surgery. Table 2 lists the locations of undescended testicles by age group. Most of the testicles were found intra-operatively in the inguinal canal (84.3%). There were no statistically significant differences (p = 0.662) in testicular location between the age groups. Concomitant diseases included Kallmann syndrome, Down syndrome, Prader-Willi syndrome, hypogonadotropic hypogonadism, hypospadias, CATCH 22 syndrome, mental retardation, and Williams syndrome.

**Table 2:** Testicle location in patients with unilateral undescended testis and concomitant diseases.

Group (unilateral UDT)	I	II	III	Total
	Age ≤1 year	1< age ≤2 years	Age >2 years	
Unilateral patient number	45	58	31	**134**
Median age (IQR), months	8.1 (7.2–11.3)	14.3 (13.3–18.2)	68.6 (41.1–90.0)	
Follow-up (IQR), months	36.1 (27.6–57.1)	36.2 (23.9–53.7)	25.2 (11.2–34.4)	
**Location**				
Internal ring and above	4 (8.8%)	7 (12.1%)	1 (3.2%)	**12**
Inguinal canal	38 (84.4%)	48 (82.8%)	27 (87.1%)	**113**
External ring	2 (4.4%)	2 (3.5%)	0 (0%)	**4**
Prescrotal	1 (2.2%)	1 (1.7%)	3 (9.7%)	**5**
Premature (gestational age < 37 weeks)	8 (17.8%)	4 (6.9%)	5 (16.1%)	**17**
**Concurrent diseases**				
Genetic disorder	0	1	2	**3**
Endocrine disease	0	0	0	**0**
Genitourinary abnormalities	1	0	0	**1**

**Figure 2:**
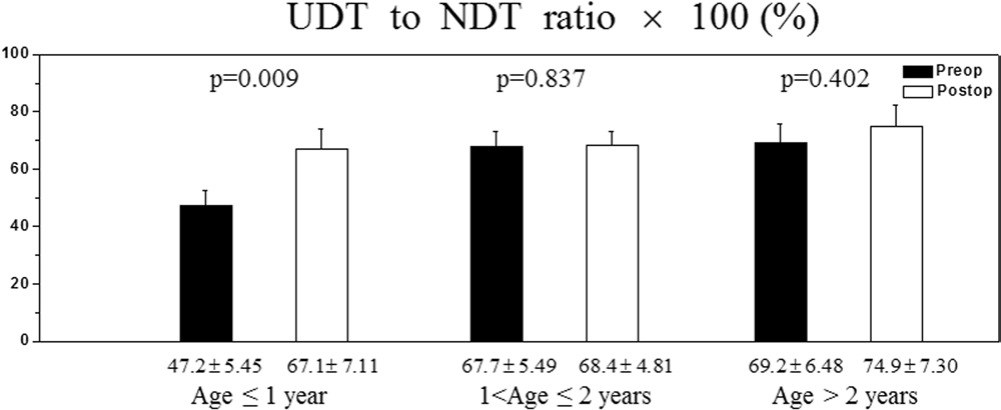
The undescended testis (UDT) to normally descended testis (NDT) ratio represents the relative size of the UDT. The p values represent results of comparison of the pre-operative and post-operative ratios.

The UDT to NDT ratio provides the relative size of the UDT compared to NDT, both pre-operatively and post-operatively (Fig. 2). In the early orchiopexy group (age ≤ 1 year), this ratio increased significantly from 47.2% at 9.2 months old to 67.1% at 44 months old (p = 0.009). In the groups with orchiopexy between 1 and 2 years of age and after 2 years of age, the UDT to NDT ratio increased after orchiopexy from 67.7 to 68.4% (p = 0.837) and from 64.4 to 88.9% (p = 0.057), respectively, with the differences not reaching the level of statistical significance. In all the groups, the volume of the orchidopexy testis remained smaller than that of the contralateral NDT.

Table 3 lists the values of testicular volume before and after orchiopexy for UDTs and the contralateral NDTs. Values in group I (age ≤ 1 year) were used as a reference. We compared several parameters between group II (1 < age ≤ 2 years) and group I and between group III (age > 2 years) and group I. Group III had the highest growth percentages of UDTs and NDTs. However, the GPR in group I (2.02 ± 0.40) was significantly higher than in group II (1.25 ± 0.13, p = 0.004) and group III (1.24 ± 0.11, p = 0.008). This result indicates that orchiopexy before one year of age is most beneficial for the growth of the undescended testicle. Nevertheless, the testicular volume of the UDT after orchidopexy remained significantly smaller than that of the contralateral NDT in all the groups.

**Table 3:** Testicular volumes of the unilateral undescended testis (UDT) and normally descended testis (NDT) before and after operation in different age groups.

Group	I	II	p	III	p
	Age ≤1 year (reference)	1< age ≤2 years		Age > 2 years	
Number of unilateral UDTs	45	58		31	
PreOP UDT volume (ml)	0.174 ± 0.018	0.234 ± 0.02	0.078	0.561 ± 0.131	<0.001
PostOP UDT volume (ml)	0.296 ± 0.028	0.306 ± 0.027	0.728	1.253 ± 0.371	<0.001
Growth Percentage of UDT	242.5 ± 72.7%	163.8 ± 18.4%	0.161	257.2 ± 61.8%	0.930
PreOP NDT volume (ml)	0.435 ± 0.026	0.393 ± 0.023	0.899	1.158 ± 0.322	<0.001
PostOP NDT volume (ml)	0.512 ± 0.034	0.517 ± 0.031	0.138	2.364 ± 0.662	<0.001
Growth Percentage of NDT	132.3 ± 9.6%	148.2 ± 10.8%	0.275	240.1 ± 59.1%	0.001
Growth Percentage Ratio (UDT/NDT)	**2.023 ± 0.404**	**1.249 ± 0.132**	**0.004**	**1.244 ± 0.113**	**0.008**

## Discussion

In current research, the UDT to NDT ratio or the alleged retained-to-scrotal testis ratio is used for direct comparison of the two testicles in a single individual^14,16^. These ratios represent the comparative size of the UDT at a particular time, either pre-operatively or post-operatively. In the present study, the UDT to NDT ratio improved after orchiopexy in all age groups. This improvement was especially significant when orchiopexy was done before one year of age (Fig. 2). A previous study also showed better volume outcomes after orchiopexy at any age, although the UDT volume remained below the normal values^17^. Kollin *et al*. reported an increase in the UDT to NDT ratio from 68 to 81% in the early orchiopexy group (treated at 9 months of age), whereas the ratio decreased in the late orchiopexy group (treated at three years old) from 68 to 56%^16^.

The postoperative UDT to NDT ratio depends on orchiopexy time and patient age. Although calculating the annual testicular growth rate can be used to eliminate the effect of follow-up duration, this method is impractical since the testicles grow very slowly in early childhood. We utilized the growth percentage ratio (GPR), a novel and comprehensive metric, to evaluate the growth of the unilateral UDT after orchiopexy. The use of the GPR is advantageous when comparing testicular growth before and after orchiopexy regardless of the follow-up duration. Furthermore, by including the growth percentages of the UDT and NDT, the GPR provides the same information as the UDT to NDT ratio. In this retrospective study with various follow-up durations, the GPR was more suitable for comparing the effects of the surgery on testicular growth. The GPR in the group of youngest children (age ≤ 1 year, 2.02) was significantly greater than the GPR in children between one and two years of age and in those older than two years. Nevertheless, the UDT grew faster than NDT in all the age groups after orchiopexy.

The two main concerns in persistent UDT are the risks of testicular malignancy and infertility. Performing orchiopexy in all prepubertal boys reduces the relative risk of testicular cancer. Orchiopexy by the age of 10 to 12 years resulted in a 2 to 6-fold decrease of the relative risk compared with orchiopexy after the age of 12 years or no orchiopexy at all^18^. In contrast, those who underwent orchiopexy at 13 years of age or later had a higher relative risk of testicular cancer^6^. However, patients who underwent orchiopexy before 10 years of age still had a 2.6-fold higher risk to develop testicular cancer than that in the general population^19^. Thus, from the viewpoint of preventing testicular cancer, the optimal timing of early orchiopexy is currently unclear.

Fertility in terms of ability to father children has not been compared between patients who underwent early and late orchiopexy. Orchiopexy in the first 18 months of life is recommended by the American Urological Association (AUA)^20^, whereas it should be performed between 6 and 12months according to the Nordic consensus^21^. According to histological evidence, the number of germ cells per tubular cross section decreases in the undescended testicle starting from week 28 of gestation^22^. Patients with early orchiopexy (before one year old) showed an improvement in tubular diameter, number of germ cells per tubule, degree of peritubular fibrosis, and presence of Leydig cells^23,24^. Our study provide solid evidence that UDT grows faster relative to the NDT when orchiopexy is conducted before one year of age.

The testicular atrophy index (TAI) used in the assessment of unilateral varicocele^25^ has also been applied for evaluating UDT in some studies^26^. TAI, defined as (contralateral testis volume - affected testis volume)/contralateral testis volume × 100, is equivalent to (1 − UDT to NDT ratio). Zvizdic *et al*. reported that TAI values of untreated UDTs ranged between 47.3 and 54.2% in 0.5–12-year-old boys^26^. However, TAI values after a long-term post-orchiopexy follow-up were not provided.

Bahk *et al*. showed that testicular size reflects the degree of spermatogenesis, testosterone level, and semen profile in adult patients^13^. However, no studies reported the correlation between these characteristics and testicular volume in early childhood. The limitations of the study include incomplete data owing to its retrospective nature and a large proportion of patients without ultrasonography follow-up. It was sometimes difficult to measure testicular volume in crying infants. Furthermore, more evidence needs to be collected by investigating the hormonal levels and fertility in these patients.

## Conclusion

Orchiopexy performed before the age of one year significantly improves the growth of the undescended testis, as assessed using the growth percentage ratio (GPR), compared to orchiopexy at an older age. The present clinical evidence suggests that orchiopexy before one year of age is optimal in terms of testicular growth, but it is still beneficial in older boys.
